# Gut microbiome signature in response to neoadjuvant chemoradiotherapy in patients with rectal cancer

**DOI:** 10.3389/fmicb.2025.1543507

**Published:** 2025-04-09

**Authors:** Tingmei Duan, Zhengting Ren, Haili Jiang, Yan Ding, Hongyan Wang, Fan Wang

**Affiliations:** ^1^Department of Radiation Oncology, First Affiliated Hospital of Anhui Medical University, Hefei, China; ^2^Department of Integrated Chinese and Western Medicine Oncology, First Affiliated Hospital of Anhui Medical University, Hefei, China

**Keywords:** rectal cancer, neoadjuvant chemoradiotherapy (CRT), gut microbiome, proteomics, 16S rRNA sequencing

## Abstract

**Background:**

Rectal cancer remains a leading cause of cancer-associated mortality, especially in advanced cases with limited treatment options. Emerging evidence suggests that the gut microbiome may influence the therapeutic efficacy of neoadjuvant chemoradiotherapy (CRT).

**Objective:**

This study aimed to explore the dynamic changes in gut microbiome composition and metabolic pathways in rectal cancer patients undergoing CRT.

**Methods:**

Paired fecal samples were collected from rectal cancer patients pre- and post-CRT. 16S rRNA amplicon sequencing and proteomics analysis were conducted to investigate microbial and metabolic alterations.

**Results:**

Significant shifts in the microbiome were observed, with *Fusobacterium*, *Subdoligranulum*, *Prevotella*, *Alloprevotella*, and *Bacteroides* being enriched pre-CRT, while *Streptococcus*, *Megamonas*, *Megasphaera*, *Escherichia-Shigella*, and *Olsenella* became dominant post-CRT. Metabolic analysis revealed upregulated carbohydrate metabolism and downregulated lipid and energy metabolism.

**Conclusion:**

These findings identify potential microbial biomarkers and metabolic pathways associated with CRT response, offering insights into personalized treatment strategies.

## Introduction

Colorectal cancer (CRC) is the third most common cancer and the second leading cause of cancer death globally, with rectal cancer accounting for one-third of these cases (Keller et al., 2020). Total neoadjuvant therapy (TNT), which includes chemotherapy and radiotherapy or chemoradiotherapy, has become the well-established standard treatment approach for patients with high-risk disease features, aiming to prevent local recurrence and distant metastases (Kagawa et al., 2024).

The gut microbiome has emerged as a critical factor influencing cancer treatment outcomes. It is well-established that commensal microbes within the lumen of the human gastrointestinal tract coevolve with the host in a mutualistic relationship (Teng et al., 2023). Growing evidence has revealed that the gut microbiome plays crucial roles in rectal cancer progression through inflammation, immune responses, and metabolic reactions (Qu et al., 2023), thereby impacting the efficacy of therapies such as CRT. However, the changes in the microbiome and proteomics associated with CRT remain poorly understood.

This study aimed to characterize the temporal dynamics of gut microbiome composition during CRT, identify potential microbial biomarkers associated with treatment response, and elucidate the underlying molecular mechanisms through integrated proteomic analysis. Using 16S rRNA amplicon sequencing on six fecal samples from three patients, we observed significant changes in the gut microbiome before and after CRT, with *Fusobacterium* showing the most notable decrease and *Streptococcus* exhibiting the most significant increase. Proteomic analysis revealed substantial alterations in biological processes, cellular components, and molecular functions, providing insights into the mechanisms underlying CRT response. These findings highlight the potential impact of gut microbiome and metabolic pathways on treatment outcomes and offer a foundation for further exploration of microbial biomarkers and therapeutic strategies.

## Materials and methods

### Patient samples

The samples and clinical information were collected under conditions of informed consent, and the protocol was approved by the Ethics Committee of the First Affiliated Hospital of Anhui Medical University Clinical Medical Research. A total of six patients with newly diagnosed pathologically proven rectal cancer who underwent CRT were enrolled at the First Affiliated Hospital of Anhui Medical University from February 2023 to September 2023. The nCRT consists of conventional radiotherapy at 45–45 Gy delivered in 25 fractions. Concurrent chemotherapy was administered using CapeOx (oxaliplatin 130 mg/m^2^ intravenous infusion every 2 weeks and capecitabine 1,000 mg/m^2^ orally twice daily, 5 days/week). Fecal samples were prospectively collected 1 day before the beginning of radiotherapy and the last day of CRT. Patients were inquired about their use of antibiotics, probiotics, and proton pump inhibitors within 2 weeks prior to fecal sampling. Fecal samples were aliquoted into approximately 1 g weight specimens. Each specimen was treated with 50 mL of 1% sodium azide solution, quenched in liquid nitrogen for 15 min, and then stored at −80°C until subsequent processing and analysis.

#### Amplicon and meta transcriptome sequencing

The genomic DNA of each fecal sample was extracted using a DNA Isolation Kit according to the manufacturer’s guidelines. DNA concentrations were determined using a Qubit fluorometer, and their integrity and size were verified via 1% agarose gel electrophoresis.

For each sample, the hypervariable V3–V4 region of the 16S rRNA gene was amplified using 338F and 806R primers. Positive amplicons with different barcodes were purified and pooled equally and then sequenced at the PTM BioLab, Co., Ltd. (Hangzhou, China) on a MiSeq platform (Illumina, San Diego, CA) using a 2 × 300 bp cartridge.

#### Analysis of 16S ribosomal RNA gene sequencing

Paired-end reads were generated and assigned to each sample based on their barcodes, then merged with the Fast Length Adjustment of Short Reads (FLASH) software. High-quality filtering of the raw tags was conducted to acquire clean tags using Qiime (Version 1.7.0). The chimeric sequences were filtered using Usearch (Uparse v6.0.307) software. Sequences with more than 97% similarity thresholds were allocated to one operational taxonomic unit (OTU) using CD-HIT (v4.6.1). Classification of representative sequences for each OTU was applied, and then the Ribosomal Database Project (RDP) classifier 2.10.1 was used to assign taxonomic data to each sequence. Phylogenetic analysis of the dominant OTUs was performed using Python Nearest Alignment Space Termination (PyNAST). A rarefaction curve was generated using the Mothur package for richness estimations of the OTUs. Alpha diversity was performed to identify the complexity of species diversity in each sample. To assess the diversity in samples for species complexity, beta diversity calculations were analyzed using partial least squares discriminant analysis (PLS-DA). The Wilcoxon rank-sum test and Welch’s *t*-test were used to compare bacterial abundance and diversity. Heatmaps were constructed based on the non-parametric Wilcoxon test (*p* < 0.05, *q* < 0.1) at the genus level. Linear discriminant analysis (LDA) coupled with effect size (LEfSe) was conducted to identify taxonomic and functional changes associated with response to chemoradiation in the gut microbiome.

#### LC–MS/MS proteome analysis and proteomic data analysis

##### Protein extraction

Tissue samples were retrieved from −80°C storage, weighed appropriately, and placed in a centrifuge tube. Phosphate-buffered saline (PBS) was then added to resuspend the bacterial pellet. The suspension was centrifuged at 15,000 × *g* for 5 min at 4°C, and the supernatant was discarded. The bacterial pellet was washed with PBS, and lysis buffer (containing 1% Triton X-100 and 1% protease inhibitor) was added at a volume four times that of the pellet. The mixture was incubated on ice for 30 min and centrifuged at 12,000 × *g* for 10 min at 4°C to remove cell debris. The supernatant was carefully transferred to a new centrifuge tube, and protein concentration was quantified using a bicinchoninic acid (BCA) protein assay kit according to the manufacturer’s instructions.

##### Protein precipitation and trypsin digestion

Equal amounts of protein from each sample were used for enzymatic digestion. The volume of each sample was adjusted to uniformity with a lysis buffer. Pre-chilled acetone was then added at a 1:1 ratio (v/v) to the sample, followed by vortex mixing. Subsequently, four times the sample volume of pre-chilled acetone was added, and the mixture was incubated at −20°C for 2 h to precipitate proteins. The samples were centrifuged at 4,500 × *g* for 5 min, and the supernatant was discarded. The resulting pellet was washed 2–3 times with pre-chilled acetone and air-dried. The dried pellet was resuspended in 200 mM triethylammonium bicarbonate (TEAB), and ultrasonication was performed to fully disperse the pellet. Trypsin was added to the sample at an enzyme-to-protein ratio of 1:50 (w/w) and incubated overnight at 37°C for enzymatic digestion. After digestion, dithiothreitol (DTT) was added to a final concentration of 5 mM, and the sample was incubated at 56°C for 30 min to reduce disulfide bonds. Following reduction, iodoacetamide (IAA) was added to a final concentration of 11 mM, and the sample was incubated at room temperature in the dark for 15 min to alkylate cysteine residues. The reaction was quenched by adding additional TEAB, and the resulting peptides were stored at −80°C for further analysis.

##### Liquid chromatography–tandem mass spectrometry Analysis

Peptides were dissolved in mobile phase A (0.1% formic acid and 2% acetonitrile in water) and separated using an EASY-nLC 1,200 ultra-high-performance liquid chromatography (UHPLC) system. Mobile phase B consisted of 0.1% formic acid and 90% acetonitrile in water. The gradient elution program was as follows: 0–62 min, 4–23% B; 62–82 min, 23–35% B; 82–86 min, 35–80% B; 86–90 min, 80% B. The flow rate was maintained at 500 nL/min throughout the separation process. Peptides separated using the UHPLC system were injected into the electrospray ionization (ESI) source of a Q Exactive HF-X mass spectrometer for analysis. The ion source voltage was set to 2,100 V, and both precursor ions and secondary fragments were detected and analyzed using a high-resolution Orbitrap analyzer. The mass range for the first-level MS scan was set to 400–1,500 m/z with a resolution of 120,000. For the second-level MS scan, the resolution was set to 15,000, and the dynamic exclusion time was set to 30.0 s to prevent repeated scanning of precursor ions. Data-dependent acquisition (DDA) mode was used, selecting the top 10 precursor ions with the highest signal intensity for fragmentation in the high-energy collisional dissociation (HCD) cell using a normalized collision energy of 28%. Automatic gain control (AGC) was set to 5e4, the signal threshold was set to 2.5e5 ions/s, and the maximum injection time was set to 40 ms.

##### Database search

The resulting MS/MS data were processed using the MaxQuant search engine (v.1.6.15.0). Tandem mass spectra were searched against the sample_specific_database.fasta (8,522 entries) concatenated with a reverse decoy database to estimate the false discovery rate (FDR). Trypsin/P was specified as the cleavage enzyme, allowing up to two missing cleavages. The mass tolerance for precursor ions was set to 20 ppm for the first search and 4.5 ppm for the main search, while the mass tolerance for fragment ions was set to 0.02 Da. Carbamidomethylation of cysteine residues was specified as a fixed modification, and oxidation of methionine and acetylation of protein N-termini were specified as variable modifications. The FDR for both peptides and proteins was adjusted to <1% to ensure high-confidence identifications.

## Results

**Figure 1:**
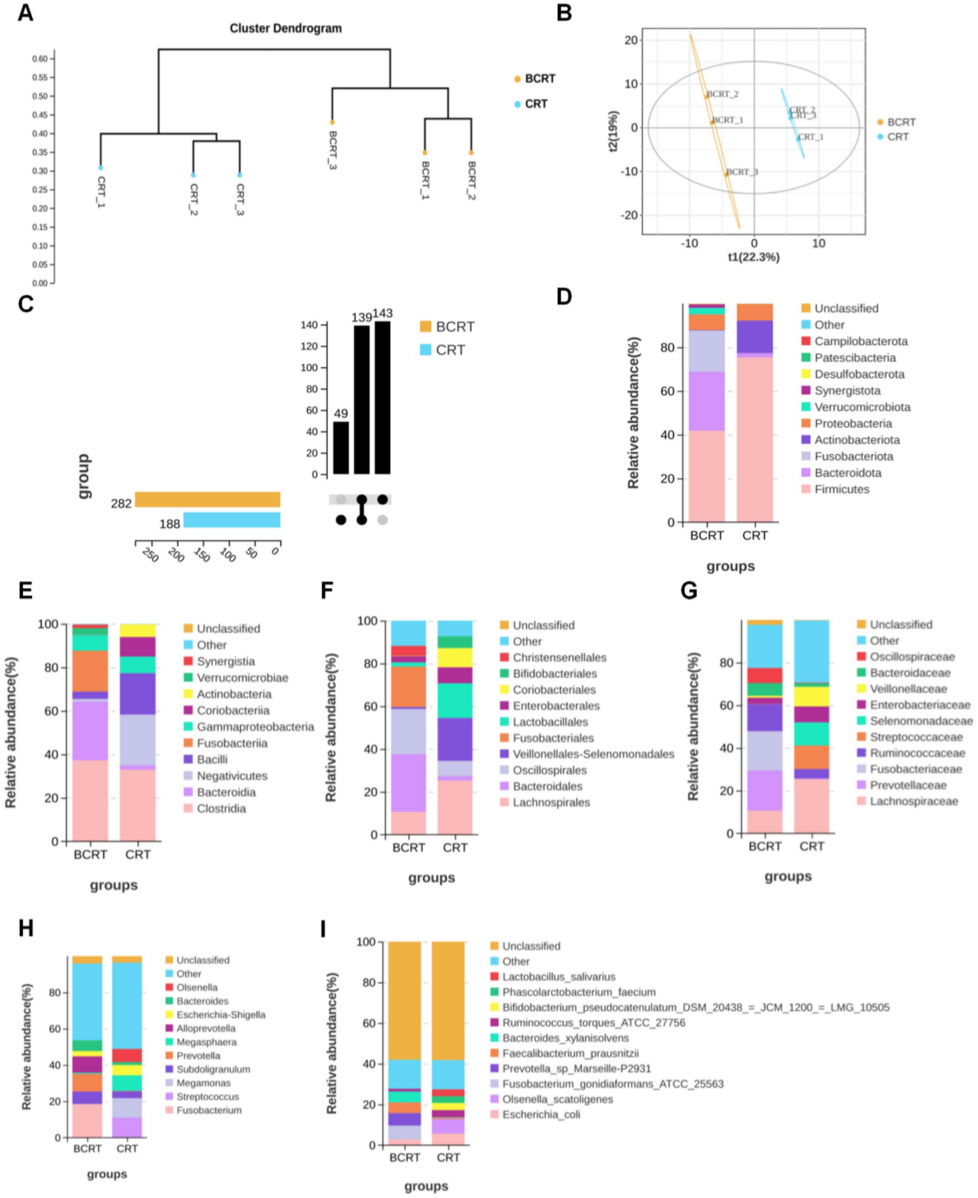
Gut microbiome in patients significantly differed under CRT. **(A)** Dendrogram of hierarchical clustering using weighted UniFrac distance at the OUT level. Each terminal branch represents a sample, and generally, samples from the same grouping are clustered into one large branch, with different groupings constituting different branches. **(B)** Partial least squares discriminant analysis (PLS-DA) of patients with BRCT (orange) and RCT (blue) (*n* = 3). Each symbol represents the data of individual patients. **(C)** The horizontal bars on the left side of the upset plot indicate the number of species in each subgroup. The bottom right is the dot matrix of the intersection part; a single node represents the information specific to the corresponding grouping, while the connecting line of multiple dots represents the information shared between groups. The top right shows the number of shared/endemic species represented by the corresponding dot matrix. **(D–I)** Stacked bar plot showing the composition of common bacteria at the levels of phylum, class, order, family, genus, and species in samples from patients with BRCT and RCT, respectively. Ranked top 10 in mean abundance among all samples were shown in detail, with other known species categorized as others and unknown species labeled as unclassified.

In the evaluation of the resulting dendrogram, the interaction-based relationships for the samples were chiefly divided into two clusters using the unweighted pair group method with arithmetic mean (Figure 1A). We explored the impact of CRT on the overall microbiome composition in fecal samples of patients by performing the partial least squares discriminant analysis (PLS-DA). The results showed that BCRT and CRT samples separated substantially (Figure 1B). We quantified 331 OTUs in both the BCRT and CRT groups, of which 139 were shared (Figure 1C). We next explored the variation in the fecal microbiome corresponding to chemoradiotherapy at different classification levels. At the phylum level, *Bacteroidetes* (26.97% vs. 1.97%) were enriched in the BCRT samples compared to the CRT samples, whereas *Firmicutes* (41.86% vs. 75.38%) were enriched in the CRT samples compared to the BCRT samples (Figure 1D). At the class level, *Clostridia* (37.29% vs. 32.96%) and *Bacteroidia* (26.97% vs. 1.97%) were enriched in the BCRT samples compared to the CRT samples, whereas *Negativicutes* (1.25% vs. 23.38%) and *Bacilli* (3.32% vs. 19.04%) were enriched in the CRT samples compared to the BCRT samples (Figure 1E). At the order level, *Lachnospirales* (10.67% vs. 25.33%) were enriched in the CRT samples compared with the BCRT samples, whereas *Bacteroidales* (26.97% vs. 1.97%) were enriched in the BCRT samples compared to the CRT samples (Figure 1F). At the family level, *Lachnospiraceae* (10.65% vs. 25.33%) were enriched in the CRT samples compared to the BCRT samples, whereas *Prevotellaceae* (18.77% vs. 0.29%) were enriched in the BCRT samples compared to the CRT samples (Figure 1G). At the genus level, *Fusobacterium* (18.44% vs. 0.03%) were enriched in the BCRT samples compared to the CRT samples, whereas *Streptococcus* (0.12% vs. 11.01%) were enriched in the CRT samples compared to the BCRT samples (Figure 1H). Moreover, *Megamonas*, *Megasphaera*, *Escherichia-Shigella*, and *Olsenella* were enriched in CRT, while *Subdoligranulum*, *Prevotella*, *Alloprevotella*, and *Bacteroides* were enriched in BCRT (Figure 1H). At the species level, *Escherichia_coli* (2.88% vs. 5.67%) and *Olsenella_scatoligenes* (0.01% vs. 7.22%) were enriched in the CRT samples compared to the BCRT samples, whereas *Fusobacterium gonidiaformans* (6.75%) and *Prevotella_sp_Marseille-P29* (6.13%) were enriched in the CRT samples (Figure 1I). Those results suggested that the microbiome at different classification levels was largely different in CRT compared to BCRT.

**Figure 2:**
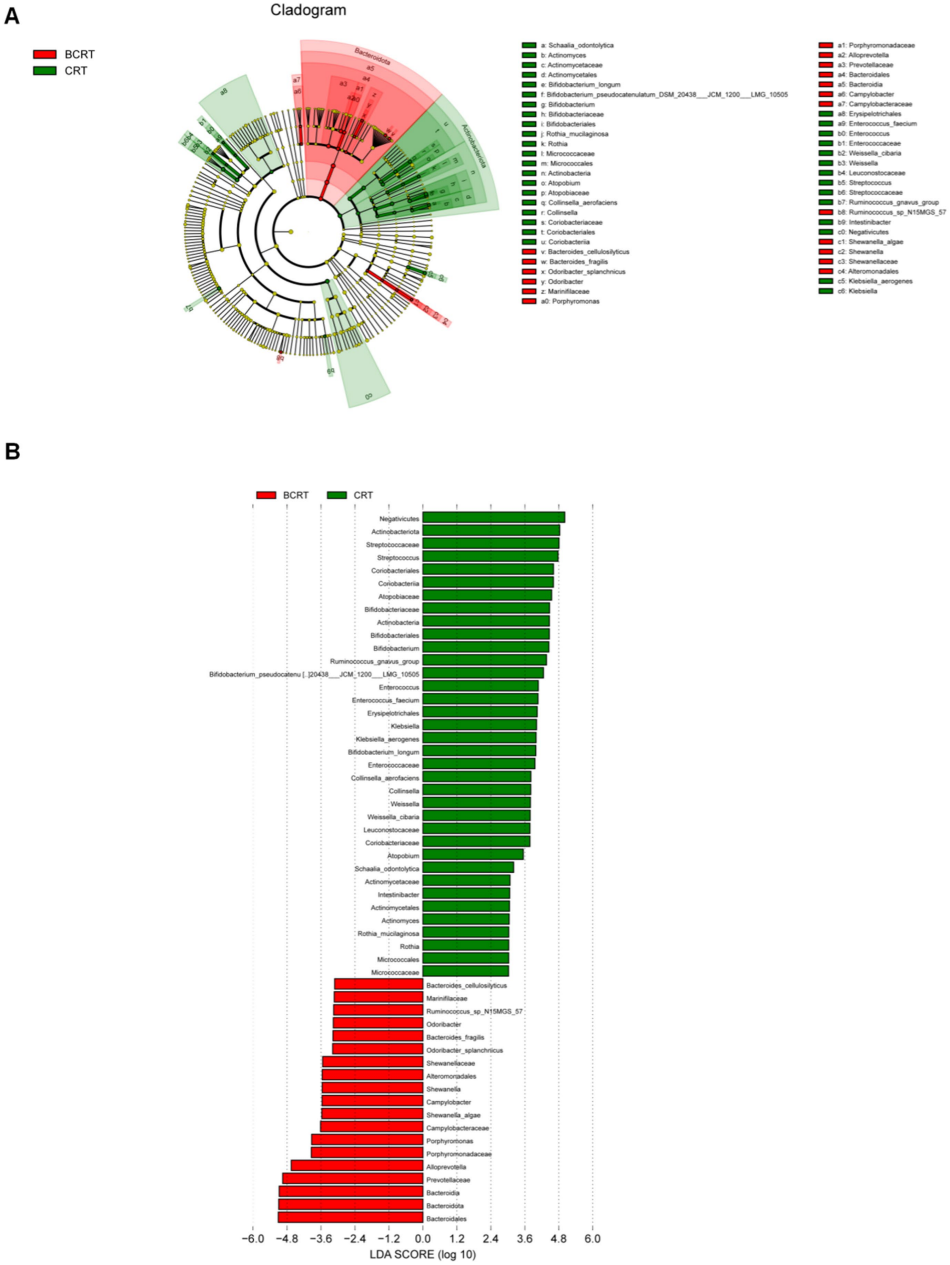
Linear discriminant analysis (LDA) integrated with effect size (LEfSe). **(A)** Genus significantly enriched (linear discriminant analysis effect: adjusted *p* < 0.05) either in samples from patients with BRCT (red) or in the RCT (green). **(B)** Cladogram plotted from linear discriminant analysis effect size (LEfSe) analysis shows the differences in the relative abundance of taxa in samples from patients with BRCT (red) and RCT (green), respectively.

To find potential biomarkers, we chose different analytical methods for screening and analyzing the indicator species between CRT and BCRT. Given that this discriminant analysis did not distinguish the predominant taxon, we performed linear discriminant analysis (LDA) integrated with effect size (LEfSe) to generate a cladogram to identify the specific bacteria associated with CRT. We screened significantly enriched microbial abundances (LDA scores (log 10) > 2.4) from the taxonomic phylum to species levels in BCRT and CRT, and 53 discriminatory OTUs were identified as key discriminants (Figure 2A). *Bacteroides* and *Alloprevotella* were significantly overrepresented [LDA scores (log10) > 3.6] in the feces of patients in the BCRT group, whereas *Streptococcus* was the most abundant microbiome in the CRT group (LDA scores (log10) > 3.6) (Figure 2B). These results indicated that CRT intervention could reduce the gut microbiome richness in patients.

**Figure 3:**
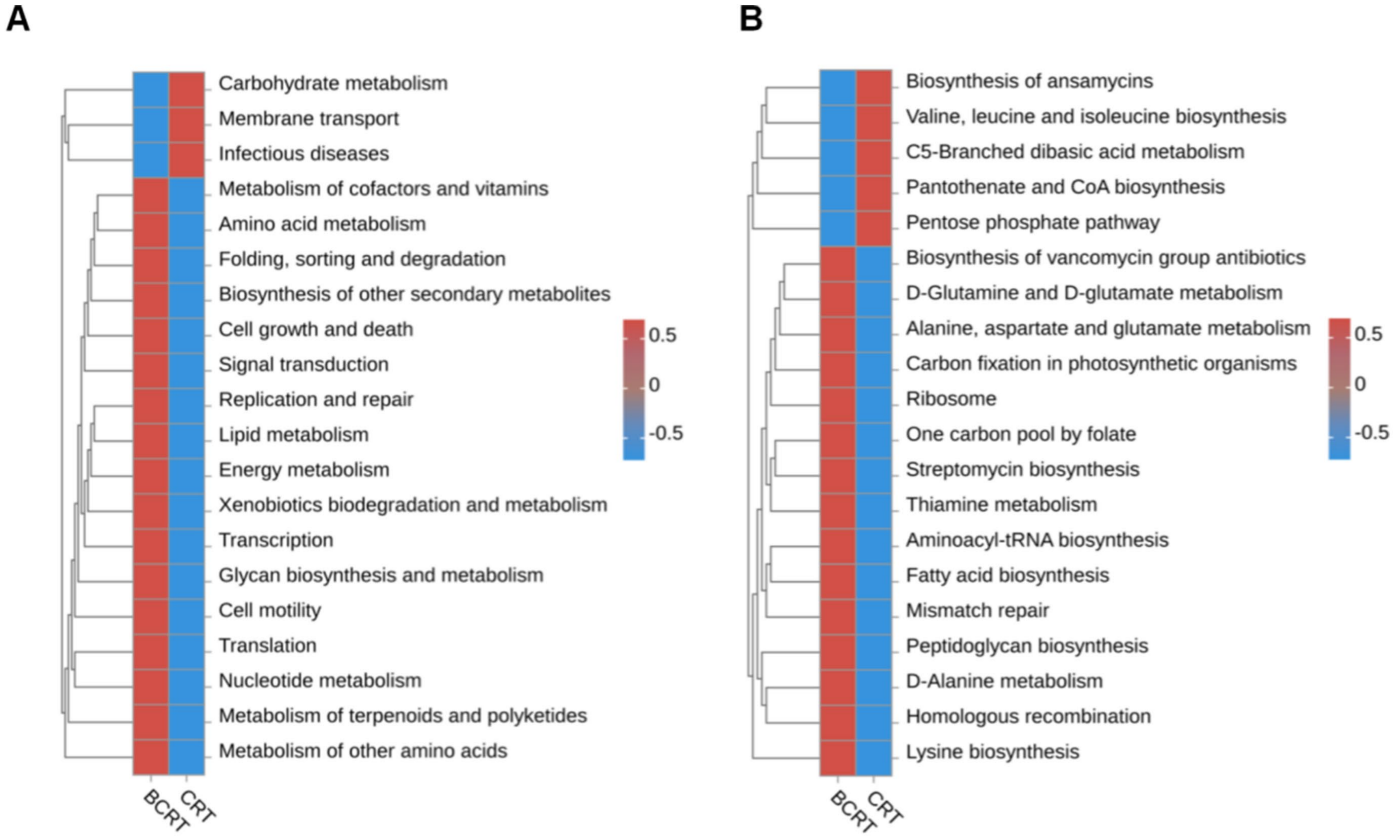
Heatmap of functional abundance. **(A,B)** The distribution of predicted functional abundance at level 2 **(A)** and level 3 **(B)** tiers in each grouping in a heatmap.

We used the OTU sequence information to find the “nearest species,” and predicted the gene information of unknown species based on the gene types and abundance information of known species, and then combined the KEGG pathway information of genes to predict the pathway of the whole community, and finally achieved the purpose of functional analysis. Compared to the BCRT group, “carbohydrate metabolism,” “membrane transport,” and “infectious disease” pathways were significantly promoted in the CRT group, while “metabolism of other amino,” “metabolism of terpenoids and polyketides,” and “nucleotide metabolism” were inhibited in the CRT group (Figure 3A). At the level of the metabolic pathway diagram, “biosynthesis of ansamycins,” “valine, leucine and isoleucine biosynthesis,” and “C5-branched dibasic acid metabolism” were promoted in the CRT group, while “lysine biosynthesis,” “homologous recombination,” and “D-Alanine metabolism” were inhibited in the BCRT group (Figure 3B).

**Figure 4:**
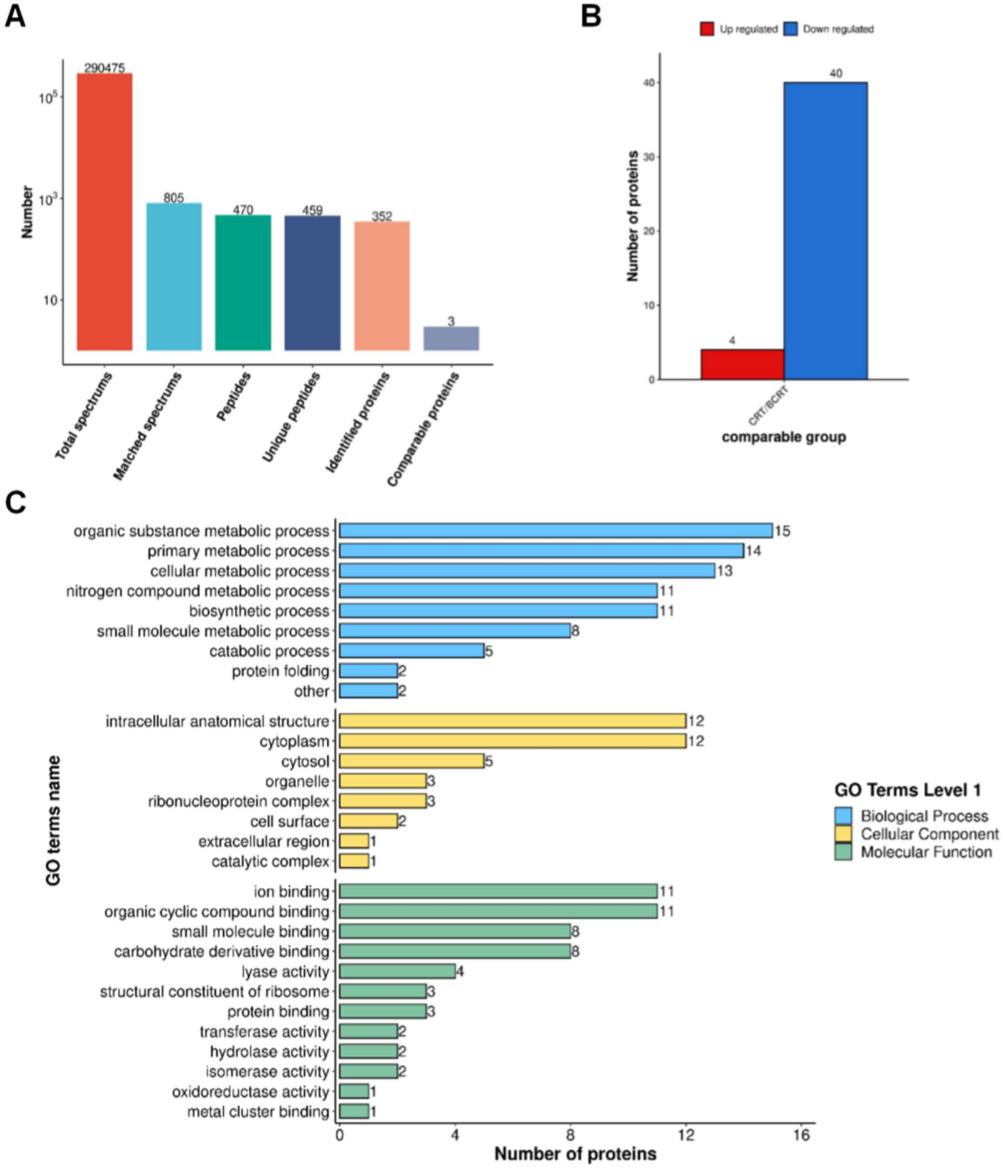
Proteomic results of fecal samples from patients in BCRT and CRT. **(A)** Protein identification by MS with spectrum extraction. **(B)** Histogram of differentially expressed proteins quantified using LC–MS/MS. **(C)** Selected proteins with a significant change in abundance in biological process, cellular component, and molecular function, respectively.

To identify proteins related to those changes, we extracted proteins from patient samples for mass spectrometry (MS) analysis. High-confidence spectra were filtered using a false discovery rate (FDR) threshold of <1%, leading to the identification of 352 proteins, among which 3 were quantified (Figure 4A). Quantified protein levels were normalized, log-transformed, and analyzed using a two-sample t-test to compare mean expression levels between the CRT and BCRT groups. The data were filtered as statistically significant when the *p*-value was <0.05, and a fold change in protein expression >1.5 was regarded as upregulation. Conversely, a fold change in protein expression <1/1.5 was regarded as downregulation (Figure. 4B). A total of 44 proteins were significantly changed, with 4 upregulated proteins and 40 downregulated proteins (Table 1). The upregulated proteins were CSV91_08665, nagA, fusA, and pyk, and the downregulated proteins mainly included rplE, gdh, and groEL. To determine the functional characteristics of the identified DEPs, Gene Ontology (GO) analysis was performed across three primary categories: biological process (BP), cellular component (CC), and molecular function (MF). Using bioinformatics tools such as eggNOG-mapper, the DEPs were mapped to GO terms based on the EggNOG database. In the BP category, the DEPs were mainly associated with “organic substance metabolic process,” “primary metabolic process,” and “cellular metabolic process,” while in the CC category, they were enriched in “intercellular anatomical structure,” “cytoplasm,” and “cytosol.” For the MF category, the DEPs were primarily linked to “ion binding,” “organic cyclic compound binding,” and “small molecule binding” (Figure 4C). Fisher’s exact test was then applied to identify statistically significant GO term enrichments, with a fold enrichment >1.5 and a *p*-value <0.05 considered significant. The analysis revealed that the DEPs were significantly enriched in pathways related to cellular metabolism, protein binding, and intracellular localization, providing insights into their potential biological roles and involvement in CRT-induced changes.

**Table 1:** Overview of 27 differentially expressed proteins identified in CRT compared to BCRT.

Protein accession	Protein description	Gene name	CRT/BCRT ratio	CRT/BCRT *p*-value	Regulated type
A0A0J6WQM0	Phenyllactate dehydratase	AB840_11920	0.001	0.001	Down
A0A3E4L847	Large ribosomal subunit protein uL5	rplE	0.001	0.001	Down
U7UES6	Glutamate dehydrogenase	gdh	0.001	0.001	Down
S7IWN6	Amino acid/amide ABC transporter substrate-binding protein	NM10_09260	0.001	0.001	Down
A0A921JUK6	NIF system FeS cluster assembly NifU N-terminal domain-containing Protein	K8V17_07285	0.001	0.001	Down
A0A133NKJ5	Chaperonin GroEL	groEL	0.001	0.001	Down
A0A173VRK7	NADH peroxidase	rbr3A	0.001	0.001	Down
A0A173XEN4	Arginine deiminase	arcA	0.001	0.001	Down
A0A173YBA9	Phosphoglucomutase	pgcA	0.001	0.001	Down
A0A173YQR3	Amidohydrolase family protein	CSV91_08665	1,000	0.001	Up
A0A174DE33	Carbon starvation protein A	cstA_2	0.001	0.001	Down
A0A174EXG9	Glucosamine-6-phosphate deaminase	nagB	0.001	0.001	Down
A0A174HZX6	Large ribosomal subunit protein bL12	rplL	0.001	0.001	Down
A0A3C0CCU1	Large ribosomal subunit protein uL11	rplK	0.001	0.001	Down
A0A174IUJ6	Phosphoglycerate kinase	pgk	0.001	0.001	Down
A0A174KAB7	Biopolymer transport protein ExbB	B5E50_00570	0.001	0.001	Down
A0A174KDP2	Ferritin OS=*Bacteroides xylanisolvens*	B5E50_17150	0.001	0.001	Down
A0A174KPV5	Transcription elongation factor GreA	greA	0.001	0.001	Down
A0A174WNA2	Triosephosphate isomerase	tpiA	0.001	0.001	Down
A0A3E5EMA8	Glutamate dehydrogenase	DW054_12070	0.001	0.001	Down
A0A175A283	Chaperone protein DnaK	dnaK	0.001	0.001	Down
A0A1H4C645	Peptidase M60 domain-containing protein	SAMN04487924_10873	0.001	0.001	Down
A0A1H4G1F2	Glutamate formimidoyltransferase	ftcD	0.001	0.001	Down
A0A1H4G585	4-deoxy-L-threo-5-hexosulose-uronate ketol-isomerase	kduI	0.001	0.001	Down
A0A1I4WKG1	Elongation factor Tu OS=*Bacteroides xylanisolvens*	tuf	0.001	0.001	Down
A0A1I5DLH7	Phosphoenolpyruvate carboxykinase (ATP)	pckA	0.001	0.001	Down
A0A1I5EKE8	Enolase	eno	0.001	0.001	Down
R7GRU0	Pyruvate kinase	BN591_01022	0.001	0.001	Down
A0A395XQI0	GGGtGRT protein	DFSSTS7063_01431	0.001	0.001	Down
A0A3C0CBX0	Glutamate dehydrogenase	DW060_05600	0.001	0.001	Down
A0A3E4LDB8	NIF system FeS cluster assembly NifU N-terminal domain-containing protein	DFSSTS7063_01432	0.001	0.001	Down
A0A3E5EH10	Branched-chain amino acid transaminase	ilvE	0.001	0.001	Down
A0A3E5EQS3	Pyruvate, phosphate dikinase	DWX30_11665	0.001	0.001	Down
A0A3E5ERF2	Elongation factor Tu	tuf	0.001	0.001	Down
A0A3R6FJR2	Malate dehydrogenase	DW060_08000	0.001	0.001	Down
A0A3R6FKH4	2-amino-3-ketobutyrate coenzyme A ligase	kbl	0.001	0.001	Down
A0A414Q040	Pseudouridine synthase	DW663_01590	0.001	0.001	Down
A0A414Q2D5	Acetyl-CoA C-acetyltransferase	DW663_00820	0.001	0.001	Down
A0A415GNJ0	Phosphoenolpyruvate carboxykinase (ATP)	pckA	0.001	0.001	Down
A0A848BSZ2	Amino-acid racemase	HF872_01435	0.001	0.001	Down
A0A9E1HNU9	N-acetylglucosamine-6-phosphate deacetylase	nagA	1,000	0.001	Up
A0A9E1HNY6	Elongation factor G	fusA	1,000	0.001	Up
A0A9E1HU24	Pyruvate kinase	pyk	1,000	0.001	Up
R6IHM0	ABC transmembrane type-1 domain-containing protein	BN533_01072	0.001	0.001	Down

## Discussion

The gut microbiome plays a pivotal role in various physiological and pathological processes, including cancer development and treatment responses (Yi et al., 2021). Emerging evidence has demonstrated that modulating the gut microbiome can significantly influence therapeutic outcomes in colorectal cancer. For instance, fecal microbiota transplantation (FMT) has shown promise in mitigating adverse effects induced by chemotherapy. Studies have reported that FMT significantly reduces phenotypes such as goblet cell loss, decreased zonula occludens-1 expression, increased apoptosis, and elevated NF-κB-positive cells caused by FOLFOX treatment (Chang et al., 2020). Additionally, novel probiotic strains, such as *Lacticaseibacillus paracasei* sh2020, have been shown to enhance the efficacy of anti-PD-1 immunotherapy by upregulating CXCL10 expression in tumors, thereby promoting CD8+ T cell infiltration and activation, reducing tumor burden, and improving the tumor microenvironment (Zhang et al., 2022). In this study, we observed significant alterations in gut microbiome composition and metabolic pathways in colorectal cancer patients undergoing CRT. These findings align with previous reports that radiotherapy and chemotherapy can profoundly impact the gut microbiome (Teng et al., 2023).

Our results found a decrease in bacterial richness, which is consistent with previous studies (Reis Ferreira et al., 2019). At the family level, we found that *Lachnospiraceae* was enriched post-CRT, consistent with its reported role in inhibiting colorectal cancer progression (Hexun et al., 2023). Interestingly, while Prevotella, a genus within the family Prevotellaceae, has been associated with a reduced risk of CRC progression and mortality (Huh et al., 2022), both Prevotellaceae and Prevotella were significantly decreased after CRT in our study. This discrepancy suggests that CRT may selectively modulate microbial populations, potentially influencing treatment outcomes.

*Fusobacterium*, a well-known CRC-associated pathogen (Ternes et al., 2020), was highly abundant pre-CRT but significantly decreased post-CRT. This reduction may reflect the therapeutic effects of CRT on targeting CRC-related pathogenic bacteria. Conversely, *Streptococcus*, which was the most enriched genus post-CRT, has previously been reported to increase in response to neoadjuvant CRT (nCRT) in patients with locally advanced rectal cancer (Yi et al., 2021). The enrichment of *Streptococcus* may indicate its potential role in modulating treatment response or immune activation during CRT.

*Bacteroides* was significantly enriched in the BCRT group, consistent with their reported role in promoting colorectal carcinogenesis. *Alloprevotella*, an oral pathogen, is less abundant in CRC patients compared to healthy individuals (Flemer et al., 2018). In our study, we observed a further decrease in *Alloprevotella* levels in the CRT group compared to the BCRT group, suggesting that it may play a distinct role in rectal cancer progression.

*Subdoligranulum*, which has been shown to increase in CRC patients (Liu et al., 2024), was found to decrease following CRT, indicating a potential reduction in harmful gut flora post-treatment. Additionally, *Megamonas funiformis*, previously implicated in CRC progression and classified under the genus Megamonas, was enriched in the BCRT group (Ren et al., 2020).

Interestingly, genera such as *Megasphaera* and *Escherichia-Shigella*, which are significantly enriched in CRC patients (Yuan et al., 2022; Shi et al., 2024), exhibited increased levels after CRT in our study, highlighting a potentially complex interplay between these taxa and treatment responses. Notably, previous studies have demonstrated that species such as *Bifidobacterium pseudolongum*, *Lactobacillus johnsonii*, and members of the *Olsenella* genus significantly enhance the efficacy of immune checkpoint inhibitors (Mager et al., 2020). We found that *Olsenella* levels were elevated in the CRT group, suggesting a potential role in promoting immune responses during treatment. These alterations in microbial genera may serve as novel biomarkers for predicting responses to CRT, offering valuable insights into the gut microbiome’s role in rectal cancer therapy.

In addition to compositional changes, we observed significant alterations in metabolic pathways. Carbohydrate metabolism was upregulated post-CRT, while lipid and energy metabolism were downregulated. These findings are consistent with previous studies showing that metabolic reprogramming, including enhanced glycolysis and altered lipid metabolism, contributes to cancer progression and treatment resistance (Chen et al., 2024). The observed metabolic shifts may reflect the gut microbiome’s adaptation to CRT-induced changes in the host environment.

Our proteomics analysis revealed differentially expressed proteins associated with CRT, including upregulated proteins such as fusA, which encodes elongation factor G (EF-G) and facilitates ribosomal translocation during translation to support microbial survival and metabolic activity, and pyk, which encodes pyruvate kinase, a key enzyme in glycolysis, indicating enhanced energy production and metabolic adaptation, while downregulated proteins such as groEL, a molecular chaperonin involved in protein folding, and gdh, which encodes glutamate dehydrogenase critical for nitrogen metabolism, suggest a reduced reliance on stress response pathways and decreased amino acid metabolism, collectively reflecting the gut microbiome’s metabolic adjustments to CRT-induced changes and providing insights into the molecular mechanisms underlying these shifts, though their full implications remain to be elucidated.

Despite these promising findings, several limitations of this study should be acknowledged. First, the relatively small sample size limits the generalizability of our results, and larger cohorts will be essential to validate these observations and explore potential variations across different populations. Second, as a single-center study, our findings may not fully reflect the diversity of gut microbiome responses in broader patient groups. Expanding to multi-center studies can help address this limitation. Third, the short follow-up period restricted our ability to assess long-term microbiome changes and their impact on treatment outcomes. Fourth, the proteomics results were not validated using orthogonal methods, which limits the robustness of our findings and calls for additional verification through complementary techniques such as targeted proteomics or functional assays. Finally, factors such as diet, lifestyle, and medication use, which are known to influence the gut microbiome, were not fully controlled and may have contributed to the observed microbial alterations. Future research should aim to overcome these limitations. Multi-center studies with larger and more diverse cohorts are needed to confirm our findings and improve their applicability. Longitudinal studies with extended follow-up periods will also be critical for understanding the long-term effects of CRT on the gut microbiome and its implications for patient outcomes. To address the lack of validation in proteomics results, future studies should incorporate orthogonal verification methods, such as targeted proteomics to confirm the differential expression of key proteins, as well as functional assays to elucidate their biological roles. Additionally, incorporating detailed data on dietary habits, lifestyle factors, and medication use will provide a more comprehensive picture of the factors driving microbiome changes during treatment. Beyond observational studies, mechanistic research is needed to clarify the causal relationships between specific microbial taxa, metabolic pathways, and treatment responses. Finally, the development of microbiome-based predictive models for CRT efficacy holds significant promise for advancing personalized treatment strategies for rectal cancer.

## Data Availability

The 16s-seq data have been made available at the NCBI SRA repository under accession number PRJNA1192684. The data and materials supporting the findings of this study are available upon reasonable request from the corresponding authors.
